# New-Onset MDA-5 Dermatomyositis in a Patient Following COVID-19 Vaccination: A Case Report

**DOI:** 10.31138/mjr.280124.nom

**Published:** 2024-03-31

**Authors:** Eleana Bolla, George E. Fragoulis, Alexios Iliopoulos

**Affiliations:** 1 Department of Rheumatology, 417 Army Share Fund Hospital (NIMTS), Athens, Greece,; 2 Rheumatology Unit, First Department of Propaedeutic Internal Medicine, Joint Academic Rheumatology Program, Medical School, National and Kapodistrian University of Athens, Laiko General Hospital, Athens, Greece

**Keywords:** anti-MDA-5 dermatomyositis, interstitial lung disease, Sars-CoV-2 infection, COVID-19 vaccination, autoimmunity induction, case report

## Abstract

Vaccination against Sars-CoV-2 has been proven to significantly reduce COVID-19 morbidity and mortality and is therefore recommended for the general population, and especially for seniors with impaired immunity. However, it is currently postulated that COVID-19 vaccines could rarely induce autoimmune diseases in previously healthy individuals. We report a case of new-onset anti-melanoma differentiation-associated protein 5 (anti-MDA5) antibody-positive dermatomyositis in a patient presenting with rash and fever following the third dose of COVID-19 vaccine. The laboratory testing revealed high titres of anti-MDA-5 antibody and chest computed tomography showed micronodular lesions and ground glass opacities bilaterally. The patient was promptly treated with corticosteroids, methotrexate, and azathioprine, and was later started on rituximab due to dermatomyositis rash exacerbation along with newly formed, diffuse skin ulcers. Our case highlights the potential immunogenicity of COVID-19 vaccines and the need for further reporting of rare rheumatic syndromes possibly related to COVID-19 disease and vaccination.

## INTRODUCTION

It is currently recognised that patients with autoimmune rheumatic diseases (ARDs) have an increased risk of morbidity and mortality following Sars-CoV-2 infection compared to the general population, prioritising them for prompt vaccination to mitigate COVID-19 risk.^1^ Although their efficacy in the general population is well documented, increasing evidence suggests that vaccines against Sars-CoV-2 could potentially cause flares in patients with already existing ARDs or even trigger new-onset ARDs in previously healthy individuals.^2^ However, a causal relationship between COVID-19 vaccination and ARD induction has not yet been established.

The anti-melanoma differentiation-associated protein 5 (anti-MDA5) antibody-positive dermatomyositis is a rare presentation of the inflammatory myositis spectrum characterised by poor response to treatment and potentially severe respiratory complications.^3^ To date, a few cases of anti-MDA-5 dermatomyositis related to Sars-CoV-2 infection or vaccination have been reported worldwide. We describe a case of new-onset anti-MDA5 antibody-positive dermatomyositis in a previously healthy individual following COVID-19 vaccination.

## CASE DESCRIPTION

The patient is a 64-year-old, female pensioner who was initially admitted to the Internal Medicine clinic of our hospital for evaluation of intermittent fever with a 48-hour periodicity, reaching 38.4^o^C and subsiding without antipyretics. The patient complained also for fatigue and weight loss which began approximately two weeks before her admission.

**Figure 1.**
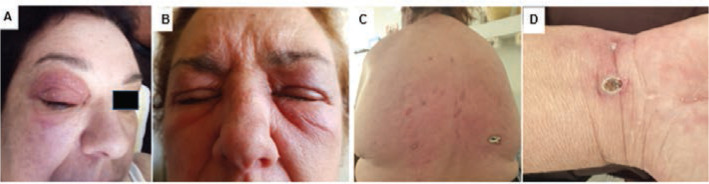
**(A)** Purplish scaling rash in the right periorbital area with associated eyelid swelling (initial manifestation). **(B)** Heliotrope erythema with associated eyelids and facial swelling (presentation on admission). **(C)** Skin ulcers on the patient’s upper trunk. **(D)** Skin ulcers on the palmar surface of the patient’s right wrist.

Fifteen months ago, she had received the third dose of COVID-19 messenger ribonucleic acid (mRNA) vaccine (BNT162b2 - Pfizer-BioNTech) and four weeks after her vaccination, she reported to have developed a purplish, oedematous, scaling rash in the right periorbital area (**Figure 1A**). During the following year, the erythema progressively spread on the left eye, face, and the upper part of her trunk. At the time, she was evaluated by ophthalmologists and dermatologists on outpatient basis, and she had received a diagnosis of chronic lacrimal gland dacryoadenitis. A biopsy of left lacrimal gland was then performed, which revealed an inflammatory infiltrate without specific diagnostic findings; fibrosis, granulomas, and necrosis were not identified and staining for immunoglobulin G4 was negative.

On admission to our hospital, she presented with painless, non-pruritic rash which covered the dorsum of her hands and elbows, her forehead, and eyelids bilaterally, along with eyelid and facial swelling (**Figure 1B**). The erythema was more prominent in the areas exposed to sunlight. She denied having recently travelled or having sick close contacts, but she had a prior history of Sars-CoV-2 infection six months ago. No relevant family history was noted. Regarding the rest of her medical history, she reported allergic rhinitis and bronchial asthma for which she was receiving bronchodilators. The patient was also receiving antihypertensives and lipid-lowering medication.

From her physical examination during the consultation from the Rheumatology Department she had a skin rash indicative of dermatomyositis, including heliotrope rash with associated eyelid and facial oedema, Gottron’s papules (erythematous, hyperkeratotic papules overlying proximal interphalangeal and metacarpophalangeal joints), and ‘’V" sign on her neck and chest wall. She also had reduced muscle strength in the right biceps (4/5) and in the quadriceps of both legs (4/5). The rest of the physical examination was unremarkable.

The initial laboratory work-up revealed mild normochromic and normocytic anaemia, slightly elevated liver enzymes, and creatine kinase (CK) (369 IU/L) with normal aldolase levels, as well as increased inflammation markers [erythrocyte sedimentation rate (ESR) 58 mm/hr, C-reactive protein (CRP) 1.7 mg/dL (normal range <0.5 mg/dL), ferritin 484.2 mg/L (normal range <204.0 mg/L)]. Her COVID-19 rapid antigen and molecular test, as well as the blood and urine cultures were negative. In addition, a wide variety of other bacterial and viral infections (M. tuberculosis, M. pneumoniae, Brucella spp, L. interrogans, B. burgdoferi, Rickettsia spp, T. gondii, T. whipplei, T. pallidum, Adenovirus, Parvovirus, Hepatitis viruses) were ruled out with respective testing. The patient’s serum protein electrophoresis was unremarkable; serum angiotensin-converting enzyme (SACE) and C3, C4 complement proteins were all within normal range. She was tested for serum autoantibodies: rheumatoid factor (RF), antinuclear antibodies (ANA), anti-double stranded deoxyribonucleic acid (anti-dsDNA) and anti-Extractable Nuclear Antigen (anti-ENA) antibodies were all negative. However, the myositis-associated panel (Euroline, Euroimmun Medizinische Labordiagnostika AG, Germany) came back with strongly positive anti-MDA5 and Ro-52, and weakly positive anti-Mi-2α antibodies. A skin biopsy was also performed showing interface dermatitis and sparse perivascular lymphocytic infiltrate. Electromyography of the upper limbs was normal, while magnetic resonance imaging of the thighs was undiagnostic.

In addition, the patient underwent a chest computed tomography (CT) scan, which showed diffuse pulmonary micronodular lesions and ground-glass opacities associated with interstitial thickening (crazy paving pattern) bilaterally and an abdomen CT scan with no remarkable findings. Pulmonary function test was unremarkable. Malignancy was ruled out by an extensive work-up, including gastroscopy, colonoscopy, abdominal ultrasound, pelvis ultrasound, digital mammography and CT of the abdomen and pelvis.

Based on the above findings, a diagnosis of anti-MDA antibody-positive dermatomyositis with associated interstitial lung disease (ILD) was made. The patient was treated with 24mg of methylprednisolone daily with gradual tapering and 15mg of methotrexate weekly.

The latter was discontinued though due to liver enzyme elevation and 50 mg of azathioprine twice daily were added. The patient did not report any respiratory symptoms during her follow-up, CK levels returned to normal and muscle strength was restored after the first week of treatment. However, in the following three months after her diagnosis, her rash worsened and spread all over her body. In addition, she gradually presented newly formed skin ulcers on the trunk, the inner surface of both her thighs, and on the palmar surface of her right wrist (**Figure 1C–D**). Given the extensive skin involvement and the patient’s intolerance to methotrexate, she received two rituximab infusions 1000mg each, 2 weeks apart, in combination with 24mg methylprednisolone and 150mg azathioprine daily. Two months after the last rituximab infusion, the patient’s dermatomyositis rash improved with attenuation of the erythema and the swelling, but the above-mentioned skin ulcers did not change significantly. Regarding the laboratory work-up, the inflammation markers including ESR and CRP, which were previously increased, returned to normal.

## DISCUSSION

We described a rare case of new-onset anti-MDA-5 dermatomyositis following the third dose of the mRNA COVID-19 vaccine. Our patient presented with rash typical of dermatomyositis (heliotrope rash, Gottron’s papules, ‘’V" sign), ILD, strongly positive anti-MDA-5 antibodies and absence of prominent muscle involvement. Although temporality does not ensure causal relationship, we discuss a possible association between COVID-19 vaccination and anti-MDA-5 antibody-positive dermatomyositis.

Since their introduction in clinical practice, vaccines against Sars-CoV-2 saved millions of lives worldwide and their administration in patients with ARDs is currently recommended by the American College of Rheumatology (ACR)^4^ and the European League Against Rheumatism (EULAR)^5^ for reducing COVID-19 risk in these patients. However, the potential induction of flares in patients with pre-existent ARDs following COVID-19 vaccination was highlighted by researchers based on data from the EULAR Coronavirus Vaccine (COVAX) registry^6^ and in the recent "ACR Guidance for COVID-19 Vaccination in Patients with Rheumatic and Musculoskeletal Diseases".^4^ In the latter, the possibility of triggering new-onset ARDs by vaccines against Sars-CoV-2 in previously healthy individuals was also discussed based mainly on data from isolated published case reports.

The anti-MDA-5 antibody-positive dermatomyositis is a rare, distinct subtype of dermatomyositis, characterised by anti-MDA-5 seropositivity, clinical absence of muscle involvement, skin ulceration, and rapidly progressive ILD.^3^ The target antigen MDA-5 is a retinoic acid-inducible gene I-like receptor that functions as a key protein sensor of viral RNA.^7^ It is suggested that viral infections, including coronavirus, activate innate immunity through the MDA-5 sensors, which form complexes with viral RNA ultimately leading to the production of inflammatory cytokines and interferons, mainly interferon type I (IFN-I). Following viral-induced cell lysis, the MDA-5/viral RNA complexes are released into the extracellular space with resultant autoantibody production against MDA-5. This proposed mechanism of anti-MDA-5 autoantibodies formation in response to Sars-CoV-2 is further supported by studies which report high prevalence of these autoantibodies in patients with COVID-19.^8^ In addition, the associations between Sars-CoV-2 and anti-MDA-5 dermatomyositis are highlighted by the hyperinflammatory response and vasculopathy observed in both diseases, as well as by similarities in their clinical manifestations, including the skin rash and rapidly progressive ILD.^9^

The COVID-19 mRNA vaccines are designed based on the mRNA that enters human cells and induces the formation of antibodies against the Sars-CoV-2 spike protein. Studies have shown that these antibodies can potentially bind to human antigens such as the MDA-5, inducing IFN-I pathway. It is also proposed that SARSCoV-2 spike protein could act as a pathogen-associated molecular pattern (PAMP) activating the NOD-like receptor protein 3 (NLRP3) inflammasome and toll-like receptor (TLR)-mediated pathways ultimately resulting in cytokine overproduction.^7^ However, the exact mechanisms linking COVID-19 vaccination to anti-MDA-5 antibody-positive dermatomyositis have not been explored sufficiently.

To date, descriptions of new-onset anti-MDA-5 antibody-positive dermatomyositis cases induced by COVID-19 infection or vaccination are not well-documented in the medical literature.^10^ Following the search strategy for writing narrative reviews recommended by Gasparyan et al.^11^ we searched for relative articles indexed in PubMed/MEDLINE and Google Scholar databases from inception to January 2024. According to our search, only a few case reports or case series including a limited number of patients have been published. This observation may be further supported by the recent recognition of anti-MDA-5 antibody-positive dermatomyositis as a distinct inflammatory myositis phenotype and the rarity of the syndrome, as well as the recent introduction of COVID-19 vaccines in the clinical practice. More specific, in a previous study^10^ four cases of anti-MDA-5 antibody-positive dermatomyositis following vaccination against Sars-CoV-2 were reported, but the authors comment that the patients might also have been genetically predisposed to the disease due to their East Asian descent. In another study,^9^ six individuals with COVID-19 vaccine-induced anti-MDA-5 antibody positive dermatomyositis were described and half of them developed rapidly progressive ILD; the authors note though that a longer follow-up period is needed to assess if patient outcomes are similar to those of previously reported MDA-5 dermatomyositis cases not associated with Sars-CoV-2 vaccination.

In conclusion, we described the a case of new-onset anti-MDA-5 antibody positive dermatomyositis in a previously healthy individual following COVID-19 vaccination. The strengths of this case report include the detailed presentation of the patient’s clinical manifestations and dermatomyositis progression over a period of approximately twenty months, along with clinical images in different stages of the disease. The case report design is a limitation; we believe though that it is important to increase reporting of relative cases in the medical literature, given the relatively recent introduction of COVID-19 vaccination in everyday clinical practice.

We would like to point out that benefits of vaccination against Sars-CoV-2 far outweigh the potential risk of systemic autoimmune disease induction. Nevertheless, rheumatologists should be aware of the possible associations between these vaccines and rare rheumatic syndromes, in our case, anti-MDA-5 antibody-positive dermatomyositis. In this context, large-scale, prospective studies are warranted to prove causality and establish surveillance for immunological adverse events induced by COVID-19 vaccination.

## Data Availability

Not applicable.

## ABBREVIATIONS

ACR: American College of Rheumatology;
ANA: anti-nuclear antibodies;
anti-dsDNA: anti-double stranded deoxyribonucleic acid;
anti-ENA: anti-Extractable Nuclear Antigen;
anti-MDA5: anti-melanoma differentiation-associated protein 5;
ARD: autoimmune rheumatic disease;
CK: creatine kinase;
CRP: C-reactive protein;
CT: computed tomography;
ESR: erythrocyte sedimentation rate;
EULAR: European League Against Rheumatism;
IFN-I: interferon type I;
ILD: interstitial lung disease;
mRNA: messenger ribonucleic acid;
NLRP3: NOD-like receptor protein 3;
PAMP: pathogen-associated molecular pattern;
RF: rheumatoid factor;
SACE: serum angiotensin-converting enzyme;
TLR: toll-like receptor
